# Our Correspondence

**Published:** 1876-04

**Authors:** 


### Our Correspondence.

SYRACUSE, N. Y., Jan. 7th, 1876.

DOCTOR:

I have taken your BISTOURY 11 or 12 years. It is a cheering, pungent, scorching, good book, and I had rather be kept out of one meal daily, than be deprived of its interesting and useful reading.

J. W. H.

---

PITTSBURG, PA., Feb. 28th, 1876.

EDITOR BISTOURY:

Can you send me an ear speculum, and what will it cost? * * * * J. D., M. D.

We do not deal in instruments, save for our own use. Send to Geo. Tiemann & Co., 67 Chatham St., N. Y., where you can obtain a nest of three ear speculums, silver-plated, for $3.00.

---

HAMILTON, ONTARIO, March 10th, 1876.

DR. UP DE GRAFF:

We have a child with hare-lip. The doctors call it double hare-lip. He is six months old, and very strong. How soon can we have him operated upon? One doctor tells us one thing and another something different. B. N.

The child is old enough now to have the operation performed. If he is in good health, go on and have the operation performed at once.

---

JORDAN STATION, Ky., Jan. 13, 1876.

DR. UP DE GRAFF:

*Dear Sir*:—As you are posted in regard to the humbugs of the day, what do you know about those "resuscitators" that are sold throughout the country, said to be good for all the ailments of man? Yours, Truly,

W. S. J.

A miserable catchpenny affair, intended for the extraction of money from the pockets of greenhorns.

---

CANISTEO, N. Y., March 7th, 1876.

*Doctor:*—I received this circular a few days since, and being very near sighted, I feel very much interested. Will you please send me your opinion of them, and very much oblige,

J. S.

The above letter contained a circular of "Dr. J. Ball's Duplex Eye Cups," for curing near and far sightedness, inflammations of the eye, cataract, &c.

We gave considerable space to this eye-cup swindle, several years ago, and had supposed that all readers of the BISTOURY were informed upon the subject; we have only to reaffirm what was then written: "All apparatus, having for its object a change in the *cornea*, with a view to correcting near sightedness, is worse than fraudulent—it is absolutely injurious, and often fatal to vision." You can no more change the form of the eye ball by atmospheric pressure, and have it remain so, than you can that of a hollow rubber ball. The moment your pressure is removed, the elastic eye ball assumes its original shape, as occurs in the case of the rubber ball. Even could the cornea be made to retain the convexity which it is claimed the eye-cups produce, *then* the difficulty would not be removed; for, as is well known by ophthalmic surgeons, "far-sightedness," or "old-sight," does *not* depend upon the flattening of the cornea, (as these eye-cup charlatans claim), but upon a hardening of the lens and consequent enfeeblement of the eye's accommodation, consequently a bulging of the cornea could do no good. Then, when they claim to cure "sore eyes," and *cataract* even, with their infernal contrivances, they utter what they know to be a willful lie. We have never seen any good follow the use of these eye-cups, but *have* seen deplorable harm, in at least two instances, where the crystaline lens was dislodged and blindness produced, through their use. Our advice is, to let them entirely alone, they can do no good and are capable of much harm.

---

Here is a letter of appreciation of the BISTOURY from Michigan, with some comments upon Dr. Ned's article:

YPSILANTI, Mich., Jan. 10th, 1876.

DEAR BISTOURY:—

I think you had better make your subscription price 60 cents, so we can make easy change. It is worth double that money to any family. Some queer articles creep into the BISTOURY, as well as in other publications; like unto the idea of a person's hair turning white in a few hours. You will join me in wondering why the boots do not change color from the same cause, and if fear changes the hair white, why jealously does not change it to green, or love to blue.

Yours, Truly,

J. W. B., M. D.

---

NEW YORK CITY, March 5th, 1876.

DR. UP DE GRAFF:

I read the BISTOURY and find advice for all manner of disease and difficulties. Will you tell me what to do for my eyes when I get lime in them? I am a plasterer, and frequently have lime fall in my eyes, often unslacked, which gives me much pain and often lays me up for weeks with inflamed eyes. What can I apply immediately, at such times, to neutralize the effects of the lime?

J. S. B.

Take sulphuric acid, one drachm. Place it in a four ounce vial, and fill it with soft water. Carry this in your pocket, and when lime comes in contact with your eye, wash it out with this solution. Then bathe your eye freely with luke warm water, or milk. This will destroy the cauterizing effect of the lime, and prevent injury to the cornea.

---

March 6th, 1876.

DR. T. S. UP DE GRAFF:

*Dear Sir.*—I have a friend whose right eye is almost sightless, and has been so from a very early age, if not from birth; he can see to distinguish large objects near him, and can see men and animals moving, but at the distance of two rods cannot tell a man from a cow, or *vice versa*. To all appearances, it is the same as the other eye, (which is very strong), the pupil dilating with it, and there is no opacity visible to the naked eye. It is more sensitive to light, causing him to squint in a bright day. Can you tell me the probable cause and if there is anything that can be done for him? Please answer in next number of BISTOURY.

Respectfully, C. A. G.

Some difficulty of the retina exists in this case. It *may* be atrophy, detachment, or effusion. Just what the trouble is, can only be discerned by means of the ophthalmoscope. Take him to an ophthalmic surgeon for examination.

---

HAWLEY, Pa., Jan. 16, 1876.

DEAR EDITOR:—

Enclosed please find 50 cts. for the BISTOURY, which is not half what you ought to charge for so valuable a journal. Please give in next issue your opinion of Cincho-Quinia, also Svapnia, two remedies now quite popular. Yours, &c.,

G. B. C.

The disposition of physicians to purchase proprietary medicines, in the form of elixirs, pills, and the various compounds recommended for all manner of ailments, is becoming alarmingly prevalent; so much so, in fact, that our regular medical journals are crowded with the advertisements of these so-called chemists, who have taken upon themselves the liberty of prescribing for the physician in advance. So many of these preparations have proved to be worthless, and nearly or quite all defective, that we look upon them as being introduced as a means of traffic rather than for any real benefit to physician or patient. The articles mentioned by our correspondent, we regard as no exception to the rule. The earlier our physicians return to the system of preparing their own formulary to suit the requirements of every individual patient, the better will it be for the reputation of the physician and the expectancy of his patients.

---

POULTNEY, N. Y., Feb. 19th, 1876.

DR. T. S. UP DE GRAFF:

*Dear Sir.*—I have a boy five or six years of age, who has an eye atrophied, blind and with a deformed pupil. This deformity is congenital. The other eye is perfect in every particular. Otherwise he a perfectly sound child without scrofulous or other taint. His mother considers it a "mother's mark." Will it eventually affect the other eye *unfavorably?* And if "yes" can any operation be performed to prevent it? I would like you to answer these questions at your own convenience. Very Respectfully,

L. D. R., M. D.

Atrophy of the eye sometimes produces sympathetic inflammation in the sound eye, when the safest plan is to immediately extirpate the offending and diseased eye. The inflammation is caused by the hardened membranes pressing upon the sensative retina or nerve, giving rise to occasional attacks of inflammation. Should this occur in your child's case, remove the diseased eye.

---

CINCINNATI, OHIO, Jan. 30th, 1876.

DR. UP DE GRAFF:

I saw a copy of the BISTOURY on the table of my physician, and read considerable in it, while waiting for him to come in. In it I saw your answers to correspondents, and thought I would drop you a line, making this inquiry: We had a traveling doctor stop here who gave a course of lectures, and then treated folks for all sorts of ailments. His great hold seemed to be capturing tape-worms, and he really did get lots of them. One man told me that he thought the doctor played some *hocus pocus* on his patients, and slipped a tape-worm into the vessel, while he was rubbing the patient's back and belly, during the operation of the cathartic. But if this is so, where did he get his tape-worm from in the first instance, for he had, as I before said, "lots of them!" * * *

J. R. K.

We, of course, know nothing of the practices of the traveling doctor referred to, and can only say, in the main, they are not to be trusted, and most of them are dishonest and tricky.

Dogs, sheep, and swine are very common subjects of tape-worm; indeed, it is rarely the case that the dog is free from this parasite. A brisk cathartic of turpentine and oil will, almost invariably, produce one, of any given length, so that it would not be very difficult for a "tape-worm doctor" to secure a considerable "stock" of the animals, to slyly slip into the vessel of his tape-worm victim.

---

NI WOT, BOULDER CO., COLO.,
Feb. 8th, 1876.

DR. UP DE GRAFF.:

Your most excellent magazine finds its way out here among the mountains, where it is appreciated quite as much as in more civilized communities. Our principal occupation out here is to hunt for gold, silver, and Indians. The latter are by far the easiest found, and give us the most trouble. But what I desired most to write about, was to ask for information about my eyes. I have been engaged in "prospecting" for gold and other precious metals, and have been caught in the mountains occasionally in deep snow-storms. In walking over the snow in the bright sunlight, I find my eyes become weak and painful, until now, I cannot bear the snow light at all. Can I do anything to relieve me of this difficulty? If so, please inform me what to do, and I will cheerfully pay for your advice. I will remain housed here until I can hear from you. J. S. L.

We send you a pair of large, blue glasses, to wear out-door in the sunlight. This is the only treatment you can give the eyes yourself. The constant exposure to the bright light, has doubtless caused congestion of the retina. Remain indoor as long as you can, then don the blue glasses.

---

NAVASOTA, TEXAS, March 9th, 1876.

DR. T. S. UP DE GRAFF:

*Dear Sir:* Your friendly letter inviting me to the Centennidelphia was received, and the compliment duly appreciated. Without snarling or showing my teeth, I never entertained any desire to attend, even before Blaine's flare up and Morton's exhibition of his old bloody shirt, and since those occurrences I feel more satisfied with my notions and my resolves. My opinion is that there will be so many *pictures* of the Rebellion, so-called, so many trophies won by the Yankees from disloyal Southerners, so many mementoes and reminiscences of the "cruel war," that every Southerner who attends will regret it and return with less feeling of fraternity, feelings more of hate than love, and fewer fascines to fill, or arches to span, "the bloody chasm." The present generation will not witness a mutual good will between the sections. I can't say a *return* or *restoration* of good will, because there never was any *de novo.* But although the great mass of the two sections have always felt envious, splenetic and antagonistic, still there are thousands on both sides who have and do cherish feelings of amity and a community of interest, and I hope you and I belong to this class. I should be pleased to re-visit Philadelphia and see what great changes and improvements have been wrought since I was there a medical student. I should like to see Elmira, your elegant Surgical Institute, and witness some of your operations, and *see you*, but I must forego it all. Business before pleasure. Several persons here will attend the great show and I hope they will have a pleasant time. * * * * * * *

Our State Medical Association will meet at Marshall, April 4th; should any of your neighbors like to visit Texas, that will be a good time to see the medical gentlemen of the State.

At some future time I will write you something for the BISTOURY.

Our winter has been so mild that in some fields several cotton stocks are yet green. Tobacco grew all winter and is blooming now. Tomatoes, and other tender vegetables, survived the few cold nights we had. Our plums have bloomed—peaches are blooming, so are dew-berries and black-berries; many forest trees are in full foliage and have been for nearly a month. I have corn up and growing in my garden, four blades high; peas in bloom—I have potatoes up; radishes, beets, &c., growing. I remain Yours Truly,

A. R. K.

The gentleman who wrote the foregoing letter is an intelligent practitioner, of Texas, and we give portions of his letter because of its indicating the fears and hopes of our southern brethren relative to our grand jubilee at Philadelphia. We are quite sure that the exhibition will not partake of the nature indicated by our friend; that nothing will be exhibited with a view to stirring up sectional hate, or wounding the pride of our sensitive southern friends. Rather will it be an exhibition of the industry, skill and capability of our vast country, and a time for all sections to come together and rejoice that we are again united, and now to remain so in peace and harmony, one with another. Contrary to the Doctor's opinion, we believe that this gathering of our people from all parts of our nation will be the means of cementing a friendship already begun, and making very many acquaintances between the sections to their mutual pleasure and benefit. Therefore, come up, Doctor, and let us have a good time at Philadelphia together.

"THE GREAT ENGLISH PHYSICIAN."—We went to hear him—went to hear the wholesome truths upon matters of hygiene, as promulgated by eminent *English* authority. Saw from his bills that he is a partner of Dr. C. W. Gleason, the celebrated lecturer of Philadelphia, who, years ago, attracted large audiences to his really useful and instructive lectures. Encouraged with the prospect of listening to an educated English physician, and he seemingly endorsed by Dr. Gleason, as before stated, we went—so did hundreds of others. We listened, and enjoyed Dr. Gleason's lectures, as recited by his "partner," almost as well as originally given by the Doctor himself. But, something about the delivery of these lectures led us to suppose that all was not right—that Dr. Gleason's partner was not master of his own language—that for a "great English physician," he exhibited a wonderful recklessness in the use of the rules of syntax; in short the deliverer of Dr. Gleason's lectures, as printed in his book, revealed the deplorable fact, that he did not understand his own language; hence we wrote Dr. Gleason for an explanation of this somewhat singular phenomenon, and behold his answer, as follows, to wit:

OFFICE OF
C. W. GLEASON, M. D.
1007 ARCH ST. PHILADELPHIA,
Feb. 23d, 1876.

*My Dear Doctor.*—I am sorry to say that I know the person you refer to as "DR. BURNER." About four years ago he purchased of my publishers one thousand copies of my book. He was then in Ohio. I have since had a number of letters of inquiry, about him, and also a few callers at my office, to know if I could endorse him, and whether he was reliable, &c. I have seen him twice in my office, and then only for a few moments. I do not know anything about him, whether a graduate or not, but my impression is very unfavorable, and I regret that he has my book for sale, or uses my name on his bills or in any other way. I have forbidden my publishers from selling any more books to him. *Rest assured he is in no way connected with me*, and I do not vouch for anything he says or does. With regard to his professional skill or ability, I know nothing. I should feel sorry to know that he had taken any liberties with my name, or had imposed upon or compromised me in the estimation of any of my old friends in your region of country. * * * *

Very Truly Yours,
C. W. GLEASON."

Now, that isn't a very flattering recommendation to come from a fellow's partner, is it? Inasmuch as he deceived the public with reference to his partnership with Dr. Gleason, we thought it barely possible that he might have meddled with his book, consequently we procured a copy, and here is a revelation! An original copy of Dr. Gleason's book has upon its title page the following:

"EVERYBODY'S OWN PHYSICIAN, OR HOW TO ACQUIRE AND PRESERVE HEALTH, WITH A VALUABLE APPENDIX, BY C. W. GLEASON, M. D."

The book sold by the "Great English Physician," has for its title page the following:

"THIRTY-EIGHT LECTURES ON HOW TO ACQUIRE AND PRESERVE HEALTH, BY C. W. GLEASON, M. D., AND H. R. BURNER, M. D., Published by *H. R. Burner, M. D.*," showing conclusively, that the "Great English Physician" appropriated Dr. Gleason's book to his own use, and claimed it as a joint production of himself and the true author. Place the two books side by side, and you will find them to be identical, save only in the title page. Attend one of the "Great English Physician's" lectures, and open Dr. Gleason's book at the page where it treats of the subject in hand for the evening, and you will discover a wonderful similarity in matter and language, save where the "Great English Physician" ventures upon a few original sentences, when his ignorance of the use of his mother tongue becomes glaringly apparent. Examine his "costly oil paintings," and you will find even those copied from the book mentioned, and are produced in the exact order in which they there occur, exhibiting a wonderful, not to say flattering approval of the sentiments of the author.

Take all circumstances into account, coupled with the evident learning of the "great English physician," and it is not difficult to imagine that he is a graduate of some English University, for we are all familiar with the uncontrovertable fact that just such talent alone can emanate from these celebrated institutions of learning. Then, were any further evidence of his skill and learning necessary, it would be found in the exhibition of his liveried valet, for it is by just such ostentatious displays that all the distinguished English physicans are known.

We congratulate our citizens,—particularly those who have so lavishly expended their money upon the "Great English Physician"—upon his advent among us, and express the hope that they may not discover, as was the case with the "Great German physician" of two years ago, that they have been sadly swindled.

